# Resting state functional connectivity in early blind humans

**DOI:** 10.3389/fnsys.2014.00051

**Published:** 2014-04-07

**Authors:** Harold Burton, Abraham Z. Snyder, Marcus E. Raichle

**Affiliations:** ^1^Department of Anatomy and Neurobiology, Washington University School of MedicineSt. Louis, MO, USA; ^2^Department of Radiology, Washington University School of MedicineSt. Louis, MO, USA

**Keywords:** blindness, human, fMRI, functional connectivity, visual cortex

## Abstract

Task-based neuroimaging studies in early blind humans (EB) have demonstrated heightened visual cortex responses to non-visual paradigms. Several prior functional connectivity studies in EB have shown altered connections consistent with these task-based results. But these studies generally did not consider behavioral adaptations to lifelong blindness typically observed in EB. Enhanced cognitive abilities shown in EB include greater serial recall and attention to memory. Here, we address the question of the extent to which brain intrinsic activity in EB reflects such adaptations. We performed a resting-state functional magnetic resonance imaging study contrasting 14 EB with 14 age/gender matched normally sighted controls (NS). A principal finding was markedly greater functional connectivity in EB between visual cortex and regions typically associated with memory and cognitive control of attention. In contrast, correlations between visual cortex and non-deprived sensory cortices were significantly lower in EB. Thus, the available data, including that obtained in prior task-based and resting state fMRI studies, as well as the present results, indicate that visual cortex in EB becomes more heavily incorporated into functional systems instantiating episodic recall and attention to non-visual events. Moreover, EB appear to show a reduction in interactions between visual and non-deprived sensory cortices, possibly reflecting suppression of inter-sensory distracting activity.

## Introduction

Abundant evidence indicates that “visual cortex”^1^ performs non-visual functions in persons who were born blind or who lost sight in infancy, (e.g., early blind, EB) (Pascual-Leone et al., 2005; Burton and Mclaren, 2008; Merabet and Pascual-Leone, 2010; Kupers and Ptito, 2011; Ricciardi and Pietrini, 2011). For example, the observation that EB can acquire Braille alexia following occipital strokes suggests that visual cortex contributes to language cognition. Such strokes do not interfere with general somatosensory awareness nor do they cause aphasia in the context of spoken language (Hamilton et al., 2000; Maeda and Yasuda, 2003). Similarly, repetitive TMS applied to visual cortex, disrupts Braille letter identification without eliminating awareness of touching a Braille field (Cohen et al., 1997).

Despite results such as those described above, the prevailing view is that visual cortex function in EB can be understood in terms of “cross-modal” processing of auditory and haptic stimuli (reviewed in Rauschecker, 1995; Kujala et al., 2000; Pascual-Leone and Hamilton, 2001; Burton, 2003; Noppeney et al., 2003; Burton and Mclaren, 2008; Merabet and Pascual-Leone, 2010; Sathian and Stilla, 2010; Dormal and Collignon, 2011; Frasnelli et al., 2011; Kupers and Ptito, 2011; Ricciardi and Pietrini, 2011; Voss and Zatorre, 2012). According to this view, visual cortex in EB contributes to a characteristic “perceptual advantage… in processing information of the intact modalities” (p. 118 in Kujala et al., 2000). Example enhanced abilities include better spatial localization of sounds (Lessard et al., 1998), sharper pitch discriminations (Gougoux et al., 2004), and finer perception of tactile grating orientation (Goldreich and Kanics, 2003). These observations suggest the possibility that visual cortex in EB becomes specialized for the analysis of non-visual modalities, possibly via enhanced functional connectivity with somatosensory and auditory cortices. That these enhanced abilities are attributable to reorganized visual cortex is suggested by the observation that TMS of visual cortex disrupts auditory spatial localization in the EB (Collignon et al., 2007). However, the “cross-modal” account implies that visual cortex in EB should exhibit enhanced functional connectivity with auditory cortex, and yet this prediction is not supported by prior resting-state functional connectivity experiments (Liu et al., 2007; Wang et al., 2008; Yu et al., 2008; Watkins et al., 2012). In fact, Liu and colleagues reported abnormally low resting state fMRI correlations between visual and somatomotor, auditory and multisensory cortices (Liu et al., 2007). The same group also reported generally reduced functional connectivity between primary visual cortex and the rest of the brain (Yu et al., 2008).

EB exhibit superior abilities in several cognitive domains not explainable in terms of “cross-modal” analysis of the sensorium. These perceptual advantages might be better explained in terms of superior working memory (Hull and Mason, 1995; Raz et al., 2007; Rokem and Ahissar, 2009; Swanson and Luxenberg, 2009; Withagen et al., 2013) and better “attentive processing of stimuli” (p. 118 in Kujala et al., 2000), whether tactile or auditory (Uhl et al., 1991; Kujala et al., 1995a,b, 1997; Röder et al., 1996; Sadato et al., 1996; Gizewski et al., 2003). Thus, activated responses in visual cortex are greatest during discrimination tasks that require attention to the stimuli. In contrast, somatosensory stimulation during non-discrimination tasks elicits weaker or even non-significant responses (Sadato et al., 1996; Burton et al., 2002a; Gizewski et al., 2003). Better working memory in EB can be demonstrated with tasks involving verbal material, pitch discrimination, and auditory spatial localization (Park et al., 2011). EB also show greater recall of rehearsed words (Amedi et al., 2003; Azulay et al., 2009) and heightened recollection of studied word lists (Raz et al., 2007). Similarly, EB retained >60% recognition of studied words after protracted delays (Raz et al., 2005) and exhibited greater recall accuracy than sighted individuals (Pasqualotto et al., 2013).

These observations suggest that sensory deprivation-induced brain reorganization (Pascual-Leone and Hamilton, 2001; Bavelier and Neville, 2002) in EB leads to a performance advantage not only in discrimination tasks involving intact sensory modalities, but also an advantage in multiple cognitive domains, e.g., enhanced recollection of studied verbal material. A general supposition is that these heightened abilities reflect re-programming of visual cortex for “metamodal” purposes (Pascual-Leone and Hamilton, 2001; Pascual-Leone et al., 2005). Supporting this view are multiple neuroimaging experiments in EB demonstrating vigorous responses throughout visual cortex to language tasks involving semantic memory (Burton et al., 2002a, 2003; Amedi et al., 2003; Noppeney et al., 2003; Raz et al., 2005; Bedny et al., 2011). Additionally, EB show evoked activity to other complex cognitive processing normally associated with visual experience such as recognition memories of human action sounds (Lewis et al., 2011) and even mirroring human actions (Ricciardi et al., 2009). It is possible that as blind engage normally “visual cortex” when performing such tasks they utilize a memory echo of sensory experiences as previously noted in sighted (Wheeler et al., 2000).

The issue is whether the functionality represented in visual cortex of EB primarily relates to attention or memory or language, or, indeed, any particular sensory or cognitive domain. To address this question, we performed a resting-state functional connectivity study contrasting 14 EB vs. 14 age/sex matched normally sighted (NS) participants. The underlying hypothesis was that reorganized resting-state networks reflect functionality represented in visual cortex of EB. We assume that the nature of this functionality can be determined by identifying which networks share correlated intrinsic activity with visual cortex in EB. Statistical significance of EB vs. NS functional connectivity differences was assessed using threshold-free cluster enhancement (TFCE) analysis (Smith and Nichols, 2009; Hill et al., 2010).

## Methods

### Participants

The EB group included 14 individuals having a mean age of 46.3, *SD* ± 11.4 (6 female) (Table 1). Blindness resulted from retinal pathology in all cases, most commonly retinopathy of prematurity. None could see patterns or objects; 6 claimed slight sensitivity to brightness and 8 had no awareness of light. All EB were Braille literate and most were fluent readers. The NS control group included 14 individuals having a mean age of 48.1, *SD* ± 11.6 (8 female). All participants provided written informed consent in accordance with the Declaration of Helsinki and guidelines approved by the Washington University School of Medicine Human Studies Institutional Review Board.

**Table 1:** **Early blind and sighted characteristics**.

**Early blind**	**Age**	**Gender**	**Preferred hand**	**Braille reading**	**wpm**	**Onset age**	**Light sense**	**Cause of blindness^a^**
EB1	41	F	LH	RH	76	0	Some	ONH
EB2	40	M	RH	BH	88.7	0	None	ROP
EB3	53	F	RH	BH	227.9	0	Some	ROP
EB4	52	M	RH	RH	113.3	0	None	ROP
EB5	31	M	RH	LH	58.7	0	Some	LCA
EB6	58	F	RH	RH	145.4	0	None	ROP
EB7	52	F	RH	BH	185.8	0	None	ROP
EB8	56	M	RH	RH	152	0	None	ROP
EB9	62	F	RH	BH	137	0	Some	GRP
EB10	54	M	RH	BH	60.2	0	Some	ROP
EB11	32	M	RH	BH	52.7	0	Some	ROP
EB12	30	M	RH	LH	60.2	0	None	ROP
EB13	55	M	RH	RH	n/a	0	None	Cataract
EB14	32	F	RH	LH	103.6	0	None	RB
Avg	46.3	6F/8M	1L/13R	3L/5R/6B	112.4		8n/4s	
SD	11.4				54.7			
**Sighted**	**Age**	**Gender**						
NS1	46	F						
NS2	42	M						
NS3	54	F						
NS4	46	M						
NS5	30	M						
NS6	57	F						
NS7	51	F						
NS8	55	M						
NS9	63	F						
NS10	50	F						
NS11	27	M						
NS12	68	M						
NS13	48	F						
NS14	36	F						
Avg	48.1	8F/6M						
SD	11.6							

a *Abbreviations: ONH, optic nerve hypoplasia; LCA, Leber congenital amaurosis; GRP, genetic retinitis pigmentosa; RB, retinoblastoma; ROP, retinopathy of prematurity*.

### Image acquisition

Magnetic resonance imaging was performed with a 3 T Trio scanner (Siemens, Erlangen Germany) equipped with a standard 12 element RF head coil. Resting-state functional connectivity was acquired using a gradient recalled echo-planar sequence (EPI) sensitive to blood oxygen dependent-level (BOLD) contrast [repetition time (*TR*) = 2200 ms, echo time (*TE*) = 27 ms, flip angle = 90°, 4-mm cubic voxels]. Whole brain coverage was obtained with 36 interleaved EPI slices (no gap) aligned parallel to the anterior-posterior commissural line. Each fMRI run included 164 volumes (6 min duration). Imaging was in a darkened room and with a blindfold covering the eyes of all participants. NS kept their eyes closed. Four runs were acquired in the EB and three in NS. We checked with the participants between runs to verify that they remained awake.

Structural images included a sagittal T1-weighted magnetization-prepared rapid gradient echo (MP-RAGE) scan [*TR* = 2100 ms; *TE* = 3.93 ms; flip angle = 7°; inversion time (*TI*) = 1000 ms; 1 × 1 × 1.25 mm voxels, 176 slices]. A T2-weighted structural image (*TR* = 8430 ms, *TE* = 98 ms, 1.33 × 1.33 × 3 mm voxels, 36 slices) was also acquired to serve as a registration intermediary between the EPI and MP-RAGE (Ojemann et al., 1997).

### Image processing

Pre-processing was performed using locally developed software (Shulman et al., 2010). Asynchronous slice timing was corrected using sinc interpolation. Correction for systematic odd/even slice-dependent intensity differences was as previously described (Hacker et al., 2012). Head motion within and across runs was corrected using rigid body realignment. There were no systematic group differences in the prevalence of head motion (*EB*: 0.25 ± 0.14 rms mm vs. *NS* 0.26 ± 0.14 rms mm; two sample *t-test*, *P* = 0.73) (Hacker et al., 2012). Transformation to Talairach atlas space (Talairach and Tournoux, 1988) was achieved using 12 parameter affine transforms obtained from sequential co-registration between an average from the first frames of each EPI run, T2-weighted and T1-weighted structural images to an atlas template (Shulman et al., 2010). To eliminate the possibility of group-dependent atlas transformation bias, the atlas representative template was generated from MP-RAGE structural images obtained in EB and NS populations, as previously described (Buckner et al., 2004). Presently reported atlas coordinates represent Talairach space as defined by the spatial normalization method (Lancaster et al., 1995).

In preparation for seed-based correlation mapping, the EPI were spatially smoothed (6 mm full width at half-maximum Gaussian kernel in each direction). Temporal low-pass filtering removed frequencies >0.1 Hz. Spurious variance was reduced using linear regression of 9 sources of nuisance waveforms and their associated temporal derivatives. These variables included the six parameters derived by head movement correction, signals in the ventricles and white matter, and a global^2^ whole-brain signal averaged over all voxels in a fixed region of atlas space (Gusnard and Raichle, 2001; Fox et al., 2005, 2009). The ventricle and white matter regressors were extracted from regions individually delineated in each participant using Analyze (Mayo Research Foundation, Rochester, MN) (Burton et al., 2012c).

### Correlation computation

Computation of Pearson correlation coefficients was for 16.4 min of resting state fMRI data between paired seed regions for each participant (temporal correlations). Concatenation of three EPI runs provided the time series^3^. Each run consisted of 164 frames minus the first 15 volumes to assure magnetization equalization and BOLD response adaptation to sequence noises at scan onsets. The signal extracted from each seed region was the average per time point across all voxels within ~1 cm^3^ spheres. A Fischer's r-to-z transformation {*Z*(*r*) = 0.5 ln [(1 + *r*)/(1 − *r*)]} improved the normality of the correlation coefficients (Jenkins and Watts, 1968). A two-sample, two-tailed *t*-test of *Z*(*r*), with assumed unequal variances, assessed whether two seed regions were coincidently active.

Temporal correlation computations included all possible combinations of 62 seed regions defined *a priori* from previous studies (Table 2). Seed regions were placed in sensory (vision, audition and touch), attention, cognitive control, and self-referential default mode networks (DMNs). Functional cortical connectivity map computations were restricted to seed regions in temporal correlation pairings with *t*-test *p*-values = 0.0005.

**Table 2:** **Talairach atlas coordinates for selected seed regions**.

**Network**	**ROI**	**Seed region**	***X*, *Y*, *Z***
Dorsal attention (DAN)	1	L intraparietal sulcus (LIPS)	−23, −66, 46^1^
	2	R intraparietal sulcus (RIPS)	26, −58, 52^1^
	3	L ventral intraparietal sulcus (LVIPS)	−24, −69, 30^2^
	4	R ventral intraparietal sulcus (RVIPS)	30, −80, 16^2^
	5	L frontal eye fields (LFEF)	−25, −12, 49^3^
	6	R frontal eye fields (RFEF)	25, −12, 50^3^
	7	L superior parietal lobule (LSPL)	−27, −54, 53^4^
	8	R superior parietal lobule (RSPL)	22, −58, 52^4^
Ventral attention (VAN)	9	L temporoparietal junction (LTPJ)	−54, −48, 37^4^
	10	R temporoparietal junction (RTPJ)	49, −50, 28^4^
	11	R superior temporal sulcus (RSTS)	55, −50, 11^4^
	12	R middle frontal gyrus (RMFG)	39, 12, 34^5^
Control	13	L anterior prefrontal cortex (LAPFC)	−35, 51, 13^3^
	14	R anterior prefrontal cortex (RAPFC)	32, 46, 16^3^
	15	L dorsolateral prefrontal cortex (LDLPFC)	−49, 16, 35^3^
	16	R dorsolateral prefrontal cortex (RDLPFC)	43, 9, 45^3^
	17	Accessory cingulate cortex (ACC)	2, 26, 30^3^
	18	Dorsal accessory cingulate cortex (DACC)	−9, 8, 40^6^
	19	L anterior inferior parietal lobule (LAIPL)	−51, −50, 43^3^
	20	R anterior inferior parietal lobule (RAIPL)	49, −48, 45^3^
	21	L anterior insula (LAINS)	−30, 17, 2^3^
	22	R anterior insula (RAINS)	29, 17, 3^3^
	23	L inferior frontal gyrus (LIFG)	−41, 6, 9^1^
	24	R inferior frontal gyrus (RIFG)	45, −3, 12^1^
Auditory	25	L TE1 (LTE1)	−41, −32, 9^7^
	26	R TE1 (RTE1)	37, −29, 14^7^
	27	L TE2 (LTE2)	−45, −35, 9^7^
	28	L TE3 (LTE3)	−60, −22, −1^7^
	29	R TE3 (RTE3)	63, −18, 5^7^
Vision	30	L primary visual (LV1)	−8, −81, 5^8^
	31	R primary visual (RV1)	11, −81, 5^8^
	32	L visual area 3A (LV3A)	−22, −85, 16^8^
	33	R visual area 3A (RV3A)	18, −88, 18^8^
	34	L visual areas V4-VP (LV4_VP)	−18, −70, −10^8^
	35	R visual areas V4-VP (RV4_VP)	23, −80, −10^8^
	36	L lateral occipital cortex (LLO)	−36, −83, 3^8^
	37	R lateral occipital cortex (RLO)	38, −80, 3^8^
	38	L middle temporal area (LMT)	−43, −69, −4^3^
	39	R middle temporal area (RMT)	48, −70, −3^3^
	40	L visual area 8 (LV8)	−27, −58, −15^9^
	41	R visual area 8 (RV8)	32, 61, −15^9^
	42	L parietal-occipital sulcal cortex (LPOCS)	−21, −68, 15^10^
	43	R parietal-occipital sulcal cortex (RPOCS)	17, −57, 10^10^
Somatosensory	44	L primary somatosensory (LS1)	−56, −18, 37^11^
	45	R primary somatosensory (RS1)	51, −18, 44^11^
	46	L BA3-trunk (LBA3_Trunk)	−31, −32, 58^11^
	47	R BA3-trunk (RBA3_Trunk)	31, −32, 58^11^
	48	L BA3-foot (LBA3_Foot)	−5, −34, 62^11^
	49	R BA3-foot (RBA3_Foot)	5, −34, 62^11^
	50	L BA2 (LBA2)	−43, −27, 44^11^
	51	R BA2 (RBA2)	43, −27, 44^11^
	52	L second somatosensory (LS2)	−35, −27, 17^12^
	53	R second somatosensory (RS2)	48, −14, 16^12^

1 Fox et al., 2005;

2 Georgieva et al., 2008;

3 Vincent et al., 2008;

4 Shulman et al., 2009;

5 He et al., 2007;

6 Dosenbach et al., 2007;

7 Burton et al., 2012a;

8 Burton et al., 2006;

9 Noppeney et al., 2006;

10 Kaas et al., 2007;

11 Burton et al., 2004;

12 *Burton et al., 2008*.

### Functional connectivity maps

Functional connectivity maps for a selected seed involved computing correlations in each participant between the 16.4 min time series in each 2 mm^3^ brain volume (voxel) and the average across all voxels in a seed region (Fox et al., 2005, 2006; Vincent et al., 2006, 2008; Burton et al., 2012c). Volumetric *Z*(*r*) maps were registered to a standard, population-average, landmark-surface-based atlas (PALS-B12) (Van Essen, 2005). The registration process involved using SureFit software to create a surface aligned through the mid-cortical gray matter in each individual (Van Essen et al., 2001; Van Essen, 2005; Van Essen and Dierker, 2007). Next, six selected landmarks per hemisphere on participant surfaces and in the average PALS-B12 atlas guided an algorithm that deformed the distribution of nodes in the native mid-cortical surface to the PALS-B12 atlas spherical surface. The computed deformation matrices directed subsequent registration of volumetric *Z*(*r*) maps to PALS-B12 surface space by aligning the coordinates of voxels to corresponding nodes with nearest neighbor coordinates (Nordahl et al., 2007). Group level surface-based functional connectivity maps for each seed were a computed random effects student's *t*-test (Holmes and Friston, 1998) of *Z*(*r*) scores at each node. Presently displayed results in PALS-B12 surface space represent two-tailed *t*-test results with probabilities of 0.05–0.0001 (*t* = ± 2.16 to 5.5, 13 df). The *t*-statistic values correspond to NS minus EB *Z*(*r*) group differences. Positive correspond to positive NS and/or negative EB correlations; negative *t*-statistic values correspond to negative NS and/or positive EB correlations.

### Assessment of statistical significance

The surface *t*-statistic maps were minimally geodesically smoothed to a FWHM ≤7.4 mm (Hagler et al., 2006). TFCE (Smith and Nichols, 2009), adapted for analysis of surface based *t*-statistic maps (Hill et al., 2010), was used to determine significant contiguous node clusters (see TFCE Implementation: http://brainvis.wustl.edu/wiki/index.php/Caret:Documentation:Statistics). Threshold-extent criteria were determined from 5000 permutations of randomly combined participants. The weight given to signal intensity and extent value were fixed at *H* = 2.0 and *E* = 1.0. We report clusters significant at *p* ≤ 0.05.

## Results

### Temporal correlations assessed in ROI pairs

**Figure 1:**
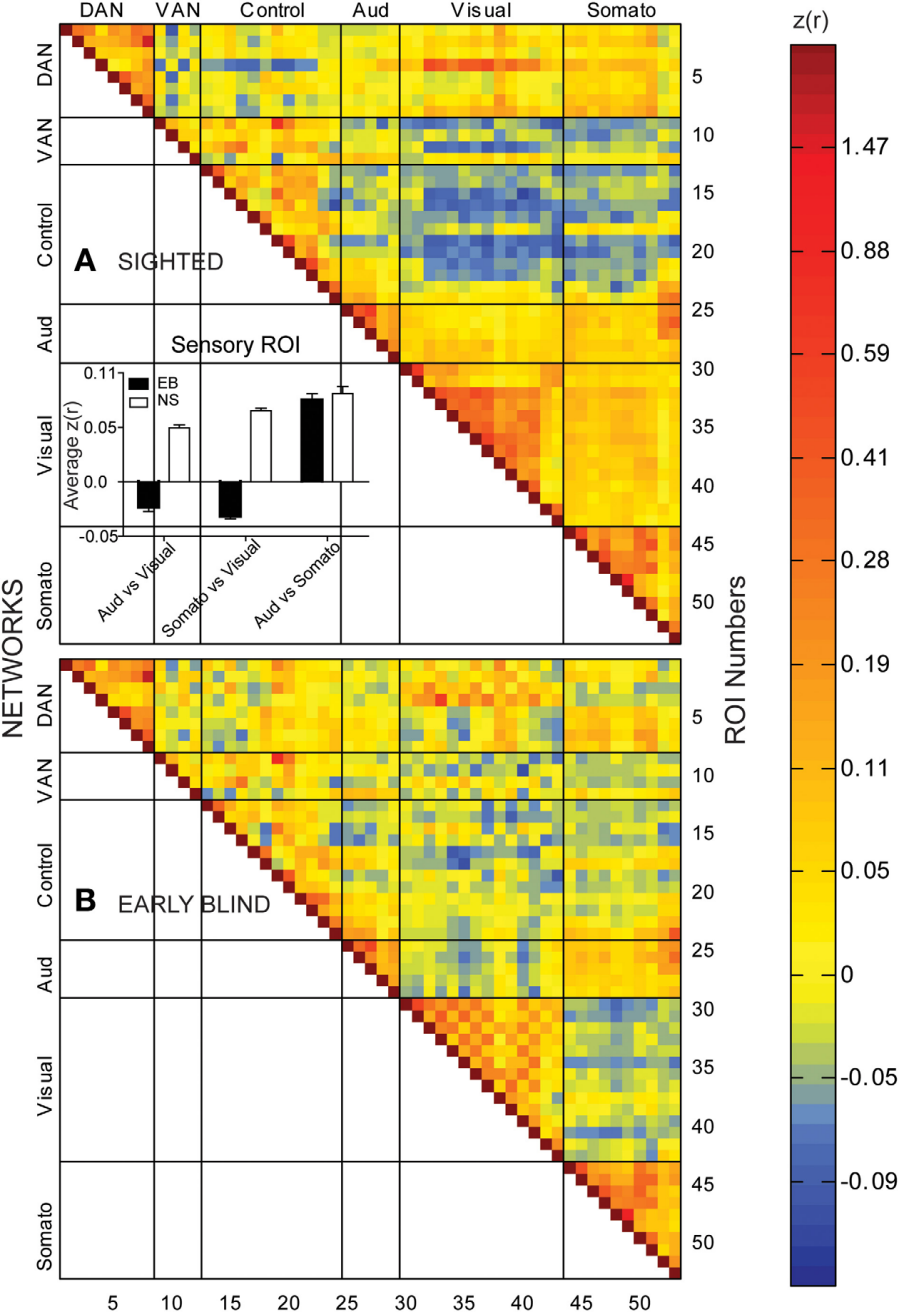
**Temporal correlation coefficient matrices computed from spontaneous, resting-state activity recorded for >16 min timelines from 53 paired seed regions in sighted (A) and early blind (B) participants**. See Table 2 for identification of ROI Numbers. Seed regions were from different networks: dorsal attention, DAN; ventral attention, VAN; cognitive control, Control; auditory system, Aud; visual system, Visual; somatosensory, Somato. Color coded scale for Pearson correlation coefficients [*Z*(*r*) values]. Insert histogram of mean and standard error of *Z*(*r*) values in each early blind (EB) and normally sighted (NS) across all seed pairings for sensory regions.

In the NS group, temporal correlations between 406 ROI pairs within and across sensory systems (auditory, visual, and somatosensory) were consistently positive. Of these, 397 (98%) pairs had *Z*(*r*) values in the range 0.01–1.01, mean 0.12 ± 0.01 s.e.m. (Figure 1A, warm hues in right lower portion of matrix). Temporal correlations between 464 ROI pairs between sensory and fronto-parietal control (FPC) and ventral attention network (VAN) ROI were shifted toward negative values, with 361/464 (78%) pairs having *Z*(*r*) values in the range −0.01 to −0.12, mean −0.05 ± 0.001 s.e.m. (Figure 1A, cold hues). These findings in NS replicate many previous resting state results (Biswal et al., 1995; Cordes et al., 2000; Lowe et al., 2000; Greicius et al., 2003, 2004; Fox et al., 2005, 2009; Fransson, 2005; Hampson et al., 2006; Vincent et al., 2006; Buckner and Vincent, 2007; Dosenbach et al., 2007; Fair et al., 2007; Fox and Raichle, 2007; Fransson et al., 2007; Vincent et al., 2008).

**Figure 2:**
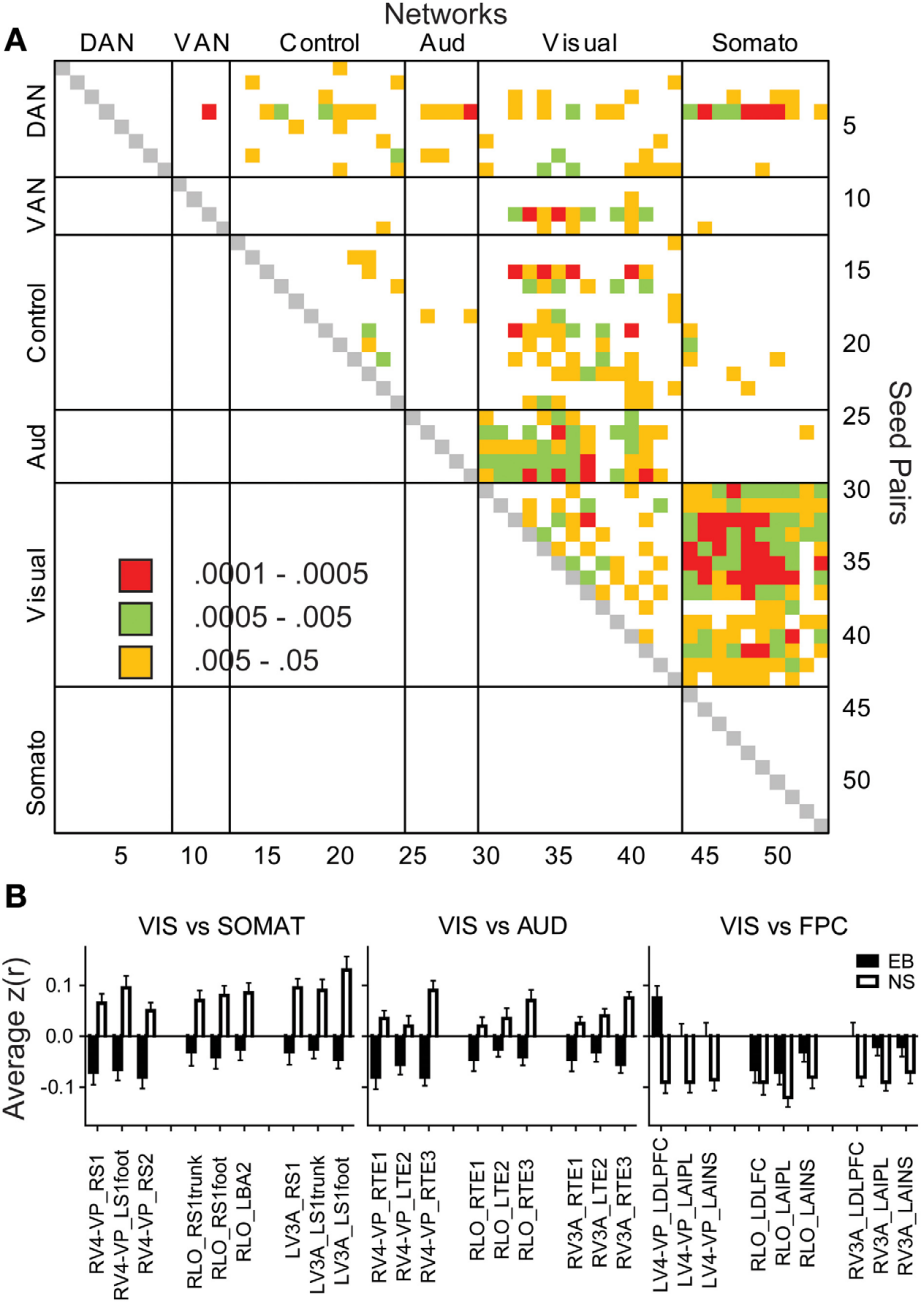
**Matrix of significant group differences in temporal correlation coefficients (A)**. Seed pairs correspond to ROI Numbers listed in Table 2. Filled cells indicate *p*-values of *t*-tests. **(B)** Bar graphs of mean and standard errors for temporal correlation coefficients in each group. ANOVAs of each bar graph had a significant group effect (*p* < 0.001) in seed pairs for visual vs. somatosensory, auditory, or control networks.

Temporal correlations in several functional systems significantly differed in the EB group (Figure 1B). Two-sided *t*-tests found significant EB vs. NS group differences in 25 pairs with *p* < 0.0005, which satisfied a Bonferroni correction (0.05/25 = 0.002). Most commonly were visual ROIs paired with ROIs in other functional systems (Figure 2A). Bar graphs in Figure 2B illustrate significant NS vs. EB *Z*(*r*) differences in selected ROI pairs.

**Figure 3:**
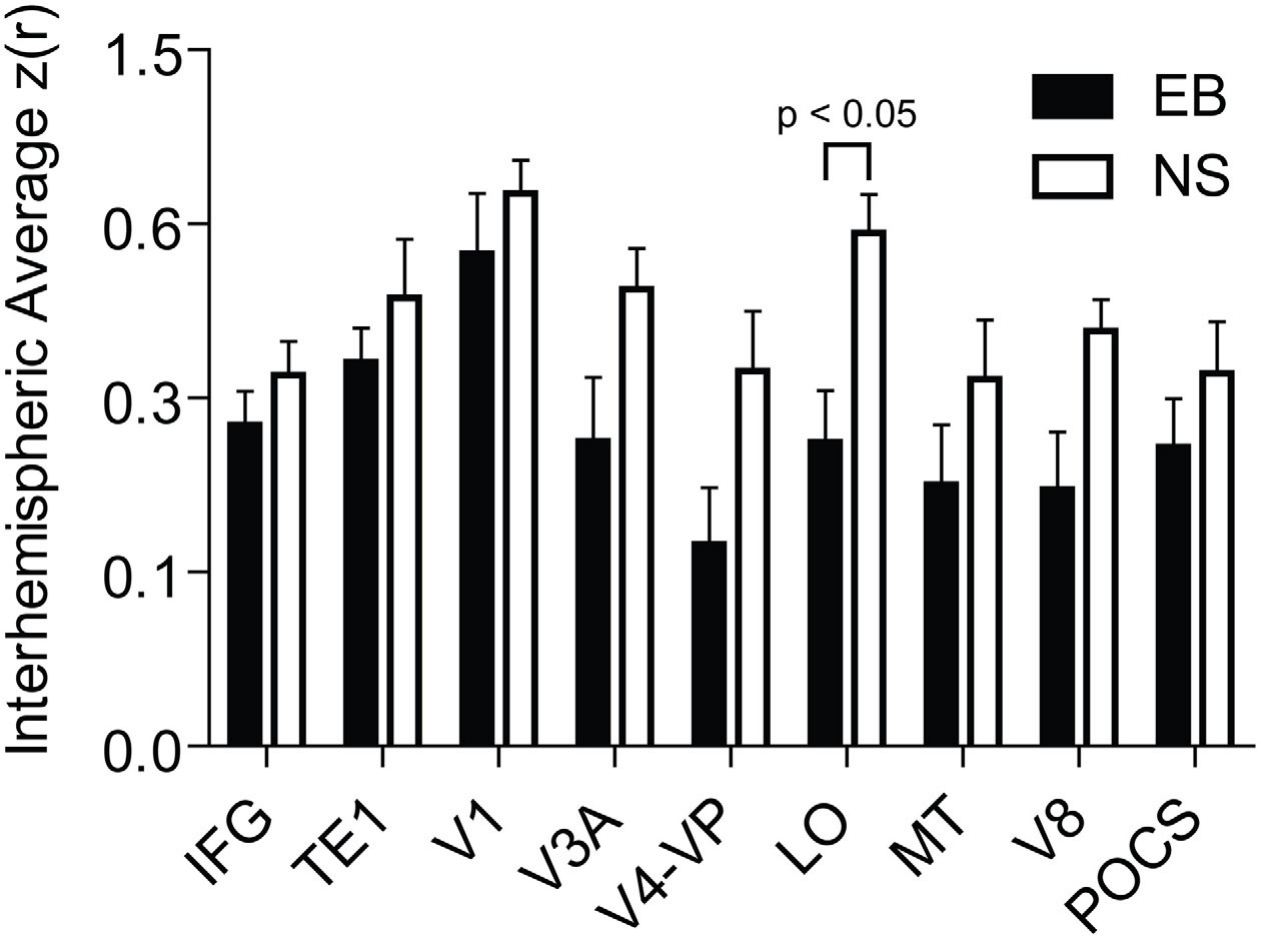
**Interhemispheric temporal correlations in homotopic seed regions in early blind (EB) and normally sighted (NS)**. Plotted data values are mean and standard error of interhemispheric *Z*(*r*) values. Abbrevations: IFG, inferior frontal gyrus; TE1, core region of primary auditory; V1, primary visual; V3A, visual area 3A; V4-VP, visual areas V4 and VP; LO, lateral occipital cortex; MT, middle-temporal area; V8, visual area 8; POCS, parietal occipital sulcal cortex.

Several features are evident in the results shown in Figures 1, 2. (i) Correlations were modestly lower within visual cortex of the EB group, more so for inter-hemispheric than intra-hemispheric correlations, leading to a checkerboard effect in Figure 1B. The greatest reduction in inter-hemispheric correlations was in higher order visual areas as opposed to V1 (Figure 3). The NS vs. EB difference in inter-hemispheric functional connectivity was significant in LO by Bonferroni corrected *t*-test. (ii) Visual cortex correlations with auditory and somatosensory cortices were consistently lower in the EB group; these differences gave rise to some of the most significant group contrasts in the present dataset. (iii) Correlations between visual cortex and nodes of the FPC network were consistently shifted toward more positive values in the EB group; 8 of these differences were significant at *p* < 0.0005 and 18 at *p* < 0.005. (iv) ROI pairs involving the DMN showed no significant group differences. The DMN ROIs are not included in the connectivity mapping results.

### Effects of early blindness assessed in seed-based correlation difference maps

**Figure 4:**
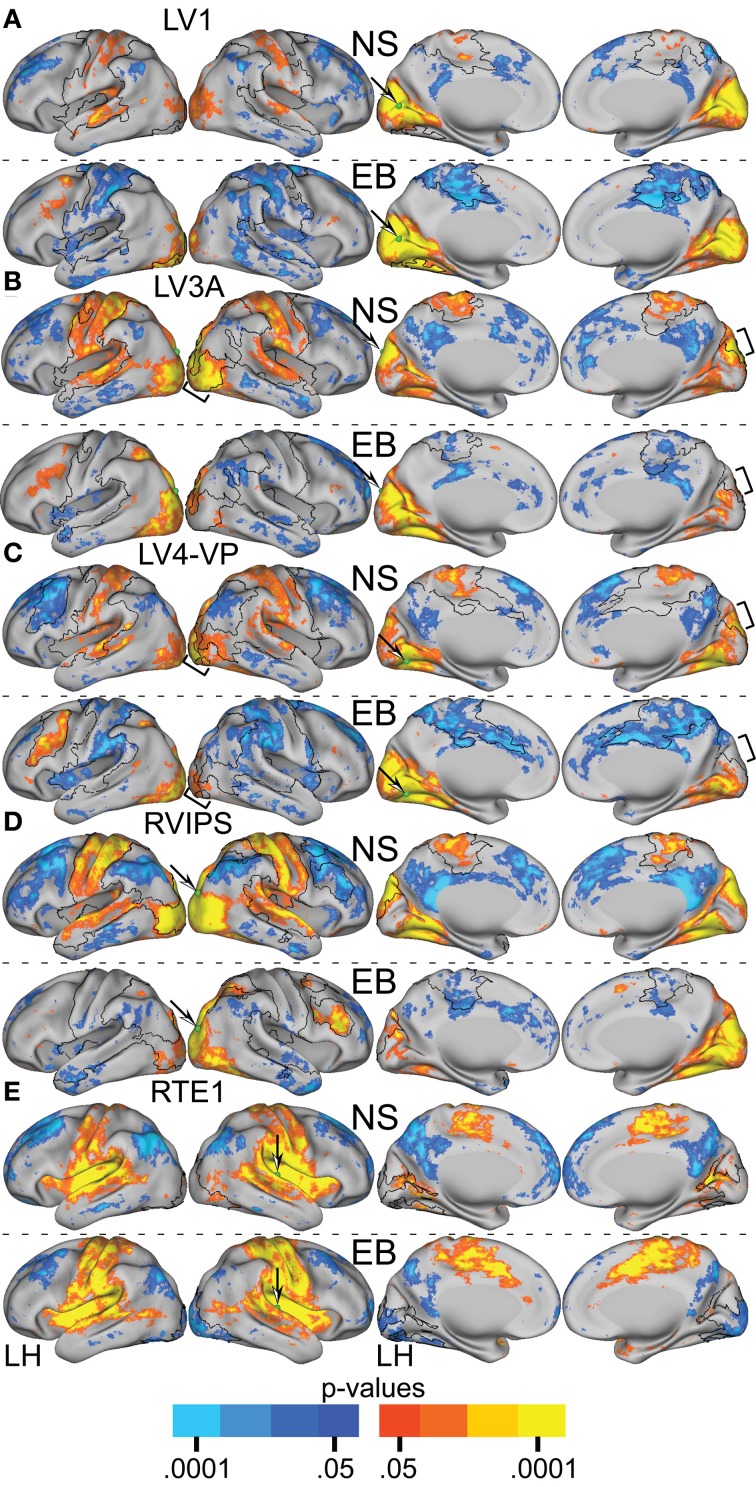
**Resting-state functional connectivity random effect *Z*(*r*) maps in normally sighted (NS) and early blind (EB) associated with selected sensory seed regions identified in Table 2**. In NS, all seed regions in visual cortex **(A–D)** showed positively correlated connectivity with other sensory cortical regions. In EB, these same seed regions showed positive correlations only within visual cortex and negatively correlated connectivity with non-visual somatosensory and auditory regions. Seed regions in non-visual sensory cortex, e.g., auditory RTE1, **(E)** similarly showed positive correlations with other sensory cortex in NS. In EB, connectivity correlations with occipital cortex were negative but positive elsewhere as in NS. An arrow points at a green sphere that marks the location of the seed region in each map. Orange-Yellow painted in cortex with positive correlations having *p*-values of 0.05 to <0.0001. Dark to light blue painted in cortex with negative correlations having *p*-values of 0.05 to <0.0001. Black borders surround significant contiguous clusters identified using the threshold-free cluster enhancement assay (see Figure 5).

Functional connectivity [i.e., *Z*(*r*) maps] were computed for all 25 ROIs yielding a group difference in temporal correlation exceeding a significance level of *p* = 0.0005 (Figure 2A). Visual cortex seeds in the NS group generated correlation maps characterized by bilateral symmetry, positive *Z*(*r*) values in somatomotor, auditory, and within visual cortex, and negative *Z*(*r*) values in dorsolateral prefrontal and supramarginal cortex (Figure 4). Differences evident in the EB group included positive rather than negative *Z*(*r*) values in left dorsolateral prefrontal and negative rather than positive values in somatomotor and auditory cortex (Figure 4). Within visual cortex, *Z*(*r*) maps for positive values were less extensive especially for higher tier visual ROI, e.g., LV3A and LV4-VP (Figures 4B,C, bracketed region). NS vs. EB differences were topographically similar across seed locations in primary (LV1) (Figure 4A).

**Figure 5:**
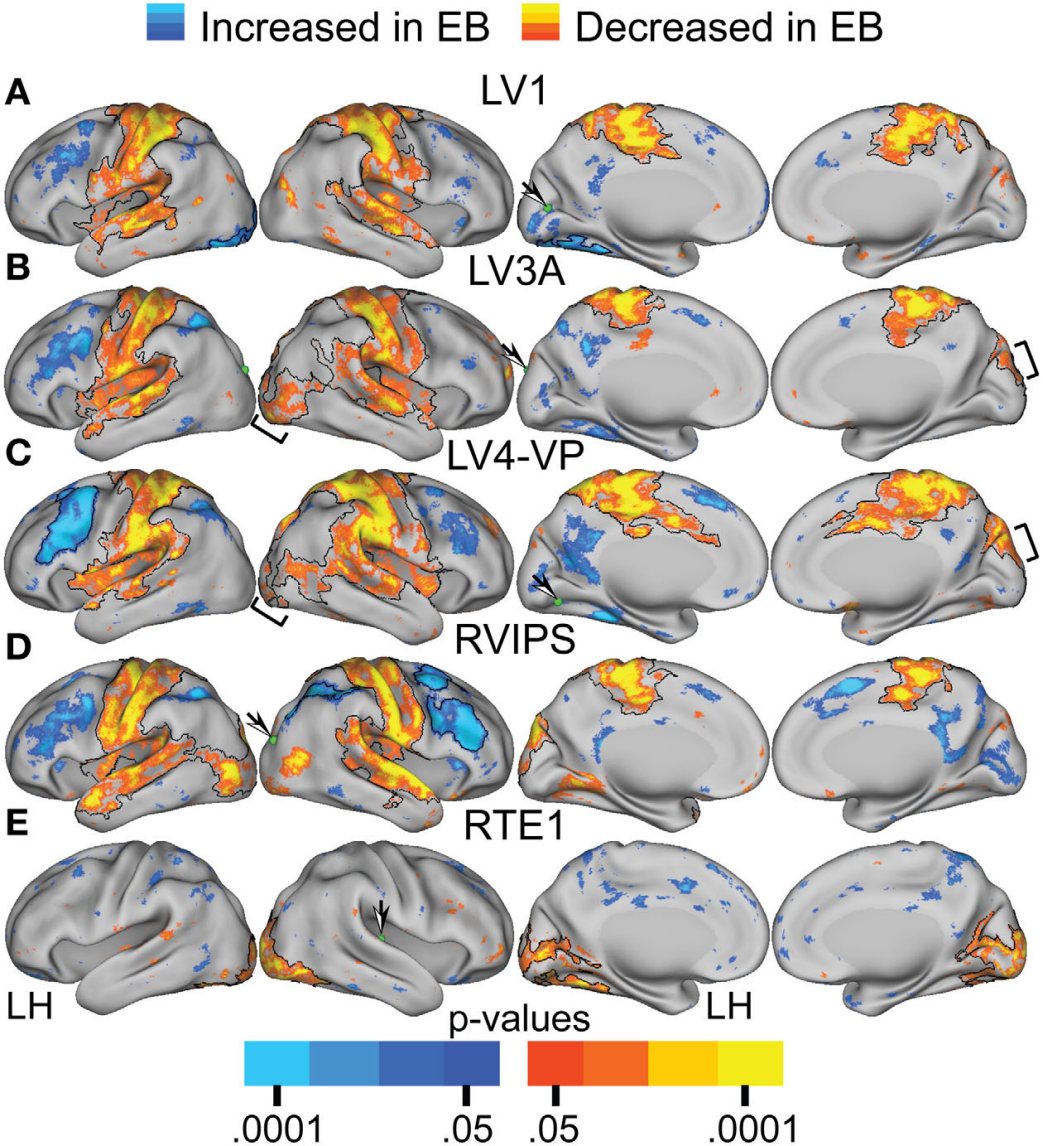
**Contrast of NS compared to EB functional connectivity based on two-tailed *t*-test for selected sensory seed regions (A–E) identified in Table 2**. The *t*-statistic maps show positive *t*-test values (orange to yellow) where NS had greater positive correlations and/or EB had greater negative correlations. The *t*-statistic maps show negative *t*-test values (dark to light blue) where NS had larger negative correlations and/or EB had larger positive correlations. Black borders surround significant contiguous clusters identified using the threshold-free cluster enhancement assay (see Methods). An arrow points at a green sphere that marks the location of the seed region in each map.

TFCE analysis of correlation maps obtained with visual cortex seeds revealed significant NS vs. EB clusters in extensive regions of somatomotor, auditory, and higher tier visual cortex as well as prefrontal and parietal regions. In greater detail, visual cortex correlations with dorsolateral frontal cortex (DLPFC), posterior intraparietal sulcal (pIPS) and left fusiform gyrus were significantly more positive in the EB group. Conversely, visual cortex correlations with somatomotor, auditory and higher tier visual (Figures 5B,C bracketed region) cortex were significantly less positive in the EB group. Figures 4, 5A–D show results obtained with 4 representative visual cortex seeds. Similar results were obtained with other visual cortex seeds (RV1, RV3A, RV4-VP, RLO, LLO, LV8, RV8).

TFCE analysis of correlation maps obtained with auditory and somatosensory cortex seeds revealed significant NS vs. EB clusters throughout visual cortex. Correlations with visual cortex were significantly more positive in the NS compared to the EB group. Figures 4, 5E show results obtained with a representative auditory cortex seed, RTE1. Other auditory cortex seeds (LTE1, LTE2, LTE3, RTE3) and somatosensory cortex seeds (LS1, RS1, LBA3, RBA3, LBA2, RBA2, LS2, RS2) yielded similar results.

**Figure 6:**
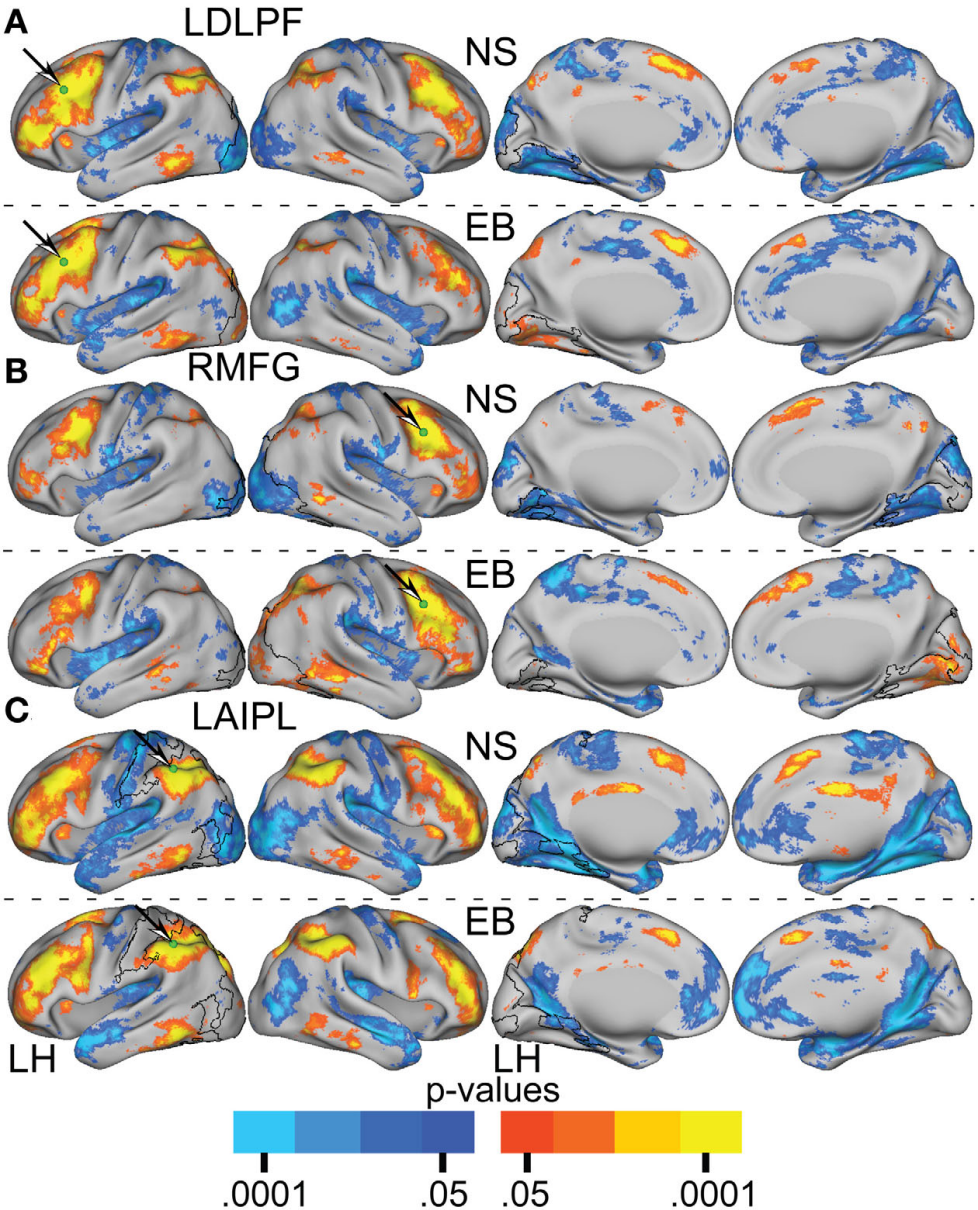
**Resting-state functional connectivity random effect *Z*(*r*) maps in normally sighted (NS) and early blind (EB) associated with selected seed regions (A–C) identified in Table 2**. A green sphere marks the location of the seed region in each map. See text for Figure 4.

**Figure 7:**
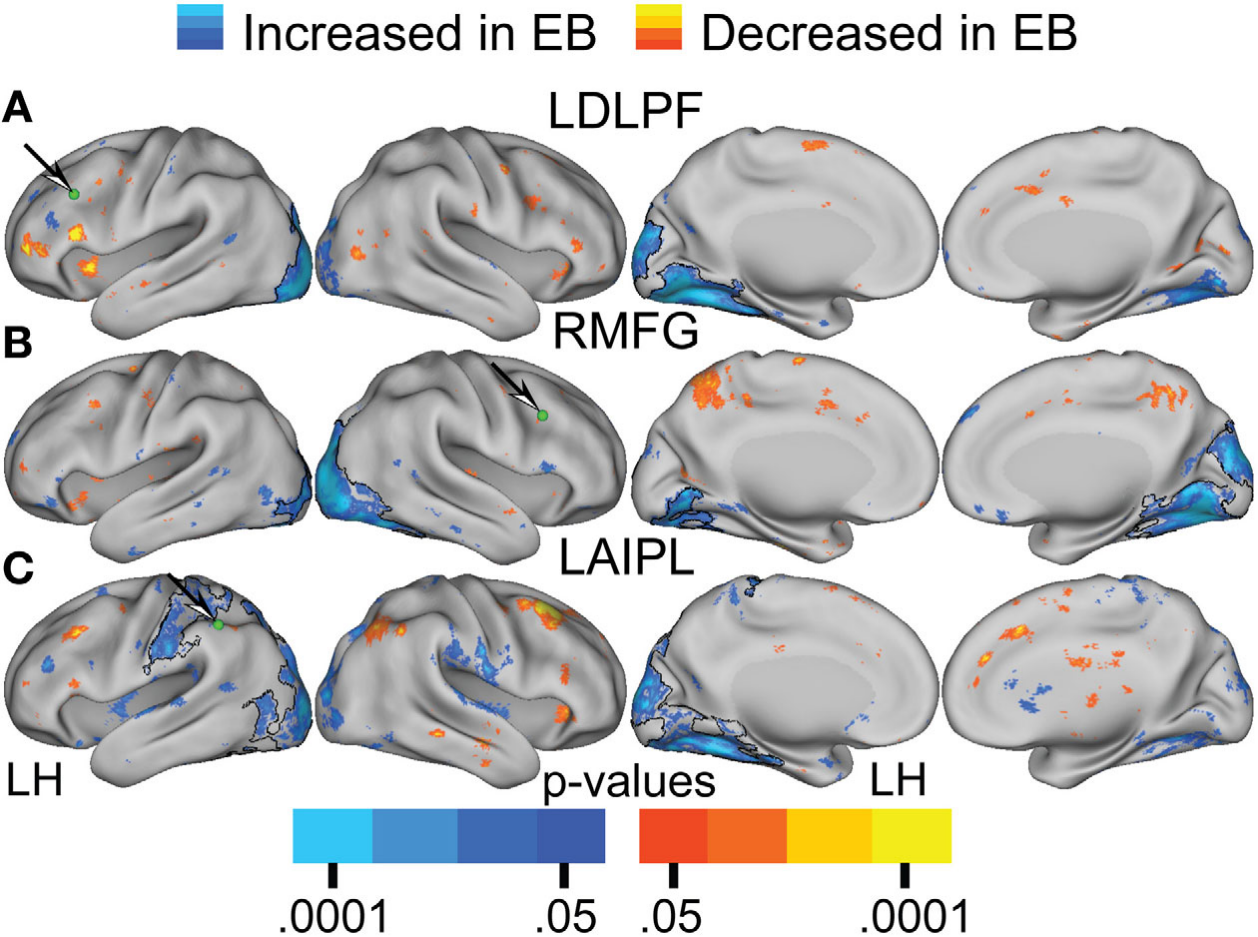
**Contrast of NS compared to EB functional connectivity based on two-tailed *t*-test for selected seed regions (A–C) identified in Table 2**. See text for Figure 5.

Figures 6, 7A–C present results for three seed ROIs in cognitive-control cortex. Each had significantly more positive visual cortex correlations in the EB as compared to the NS group. The latter effect was most prominent in extra-striate visual areas, i.e., areas V2 and V3/VP rather than V1. For the seed in left anterior intraparietal sulcus (LAIPL, Figure 6C), significantly more positive correlations extended to areas in infero-temporal cortex, probably corresponding to the V4 and V8 areas. Additional regions of significant difference included portions of parietal cortex and posterior temporal cortex in a distribution corresponding to the posterior components of the dorsal attention network (DAN). TFCE analysis of correlation maps obtained with cognitive-control cortex seeds revealed significant NS vs. EB clusters throughout visual cortex (Figures 7A–C).

## Discussion

### Overview

EB compared to NS had: (1) decreased functional connectivity between visual and somatosensory or auditory cortices; (2) inter-hemispheric visual cortex temporal correlations of lower magnitude in higher tier visual areas; and (3) increased functional connectivity between visual cortex and regions in frontal and parietal cortex associated with cognitive control.

### Sensory network functional connectivity

The “cross-modal” view of functional reorganization in EB implies greater functional connectivity between visual cortex from non-deprived sensory cortices (Wittenberg et al., 2004). Results obtained using dynamic causal modeling (DCM) support the “cross-modal” view (Noppeney et al., 2005; Ptito et al., 2008; Klinge et al., 2010; Bedny et al., 2011; Collignon et al., 2011, 2013; Kupers and Ptito, 2011; Ma and Han, 2011; Park et al., 2011; Ricciardi and Pietrini, 2011; Leo et al., 2012). These DCM results are consistent with fiber tracings in primates (Falchier et al., 2002; Rockland and Ojima, 2003) and diffusion tensor tractography data in humans showing fiber tracts between auditory and visual cortex (Beer et al., 2011). However, DCM is limited to a few ROIs at one time, selected *a priori* to study directed influences. Therefore, DCM studies, by design, cannot detect spatial reorganization in an unbiased fashion. Also, DCM targets “effective connectivity,” i.e., correlated responses induced by tasks or exogenous stimuli (as in the TMS study of Wittenberg et al., 2004). Functional connectivity based on embedded “rest” intervals is also subject to contamination from persistent responses evoked by task paradigms. Unfortunately, results obtained both by DCM and correlation analysis in the context of task performance are often referred to as “functional connectivity” (e.g., Bedny et al., 2011; Collignon et al., 2011). Such results are not directly comparable to true resting state results, as reported here.

The presented results indicate that EB compared to NS show decreased resting state functional connectivity between visual and somatosensory/auditory cortex, which is contrary to expectations based on a “cross-modal” hypothesis. Prior fMRI studies of resting-state activity also indicate decreased functional connectivity between visual and non-deprived sensory cortex (Liu et al., 2007; Yu et al., 2008). These resting state results are concordant with diffusion tensor imaging data showing absent/reduced axonal connectivity between visual and other sensory cortices (Shu et al., 2009).

The physiological substrate supporting enhanced EB visual cortex responses to non-visual tasks still needs identification. One possibility is through intra-visual cortex connections. Most extra-striate areas are multisensory in sighted individuals. In EB, non-visual perceptual tasks activate extra-striate regions that, in NS, typically respond to visual stimulation. Examples include lateral occipital cortex activation in response to tactile object recognition (Amedi et al., 2001, 2005, 2010; Pietrini et al., 2004); medial temporal area (MT) responses to perceived motion of non-visual stimuli (Hagen et al., 2002; Poirier et al., 2006; Ricciardi et al., 2007; Sani et al., 2010), and superior occipital cortex responses to spatial localization of sounds (Gougoux et al., 2005; Collignon et al., 2007, 2011, 2013) or touch (Ricciardi et al., 2006; Bonino et al., 2008). These cross-modal responses can be mediated by existing connections, as shown by reversible cross-modal activation in visual cortex of NS volunteers after relatively short periods of blindfolding (Kauffman et al., 2002; Weisser et al., 2005; Merabet et al., 2007, 2008; Lazzouni et al., 2012). Consequently, cross-modal activity in visual cortex of EB possibly involves native connections. However, like Yu and colleagues (Yu et al., 2008), we observed generally reduced intra-visual cortex functional connectivity in EB. The enhancement of cross-modal activity might then be a consequence of excitability changes resulting from visual deprivation, which even short periods of blindfolding elicit in NS (Boroojerdi et al., 2000, 2001; Merabet et al., 2008). Thus, cross-modal reorganization of visual cortex in EB is possibly a strengthened experience-dependent plasticity. Alternatively, these effects might arise from deprivation-induced developmental changes, not dependent on experience or skill acquisition (Bavelier and Neville, 2002). Adjudicating between these alternatives will require animal experiments.

The significance of reduced interhemispheric functional connectivity in higher-tier visual areas is uncertain. Similar findings were reported in patients with anophthalmia (Watkins et al., 2012). These results coincide with evidence of reduced white matter in the ventral splenium of EB (Shimony et al., 2006). Less inter-visual connectivity might explain previously described differences between EB and sighted in spatially discriminating tactile inputs (Röder et al., 2004). EB were better and faster than sighted at detecting the temporal order between two successive tactile stimuli applied to hands crossed over the midline (Röder et al., 2004). In sighted persons, vision subjugates localization of tactile events to a visual space (Eimer, 2004). Consequently, touching crossed hands in sighted provoked slower reactions from a conflict between visual and hand somatosensory space. Reaction times were faster in EB with only a tactile, proprioceptive coordinate space. Potentially further reducing confusion might be lower inter-occipital functional connectivity between higher tier visual areas like those in the superior occipital gyrus that serve tactile spatial working memory in EB (Bonino et al., 2008).

### Visual to cognitive network functional connectivity

Blindness requires greater reliance on remembering. Even blind children show compensatory, adaptive strengthening of memory skills (Withagen et al., 2013). These effects suggest long-term changes in synaptic efficacy. A consequence might be the finding of increased functional connectivity between visual and dorsolateral frontal cortex in EB for a persistently used network (Dosenbach et al., 2007; Fair et al., 2007; Lewis et al., 2009). The dorsolateral prefrontal cortex is a known contributor to recognition memory involving familiarity and remembering (Iidaka et al., 2000; Mcdermott et al., 2000; Gold and Buckner, 2002; Gallo et al., 2010). A further implication of increased functional connectivity with dorsolateral prefrontal cortex is that visual cortex contributes to memory processes in EB. A finding consistent with this notion is activity throughout visual cortex in EB for semantic tasks that engage lexical memory (Burton et al., 2002a,b, 2003). As suggested in a previous study involving recognition memory for studied words in EB, visual cortex activity possibly generates a recollection heuristic that augments remembering as opposed to familiarity (Burton et al., 2012b).

A prior functional connectivity study suggested that left visual cortex specifically contributes to semantic and lexical processing in EB, based on greater connectivity with left inferior frontal cortex language areas (Bedny et al., 2011). The current study observed neither a left hemisphere functional connectivity bias nor a significant TFCE cluster confined to left inferior frontal language areas. This negative result is consistent with diffusion tensor tractography evidence of balanced bilateral inferior frontal-occipital (IFO) fasciculi (Shimony et al., 2006; Shu et al., 2009).

The notion of a language cognition role for visual cortex in EB was invoked to explain the results of TMS experiments (Cohen et al., 1997). Further support was obtained from the observation that Braille alexia may follow visual cortex strokes (Hamilton et al., 2000; Maeda and Yasuda, 2003). An alternative explanation is that the visual cortex lesions might have disturbed attention to memory processes. Thus, patients with Braille alexia might be unable to attend to tactile features during fluent braille reading, which is an attention demanding perceptual task focused on lateral shearing produced by the pattern of dot-gaps within braille cells (Millar, 1987; Pring, 1994).

Exceptional “attentive processing of stimuli” (p. 118 in Kujala et al., 2000) probably reflect learned behaviors in EB. Visual cortex activation during auditory spatial localization tasks (Kujala et al., 2000; Collignon et al., 2006; Weaver and Stevens, 2007) have been conventionally interpreted as reflecting “cross-modal” processes, but they might be alternatively interpreted as reflecting enhanced top-down attentional mechanisms. EB show activation of visual and parietal attention areas during a tactile working memory task (Burton et al., 2010). These adaptive behaviors in EB might alter synaptic efficacies that result in increased functional connectivity between visual and cortical regions concerned with attention. Present results supporting this interpretation include increased functional connectivity between visual and anterior inferior parietal cortex, superior part of the supramarginal gyrus, anterior insula, and posterior middle frontal gyrus. Anterior inferior parietal cortex contributes to attention modulated task switching (Vincent et al., 2008). Anterior insula serves cognitive switching in conflict situations (Roberts and Hall, 2008; Sridharan et al., 2008). Supramarginal gyrus is active during attention to memory (Cabeza, 2008; Cabeza et al., 2008; Ciaramelli et al., 2008; Olson and Berryhill, 2009). Similar supramarginal gyrus responses can be seen in responses to working, long-term and episodic memory paradigms (Buckner and Wheeler, 2001). Posterior middle frontal gyrus responses are correlates of orienting to salient, yet unexpected stimuli (Corbetta and Shulman, 2002).

## Conclusions and study limitations

A crucial accommodation to blindness is reliance on memory and facile attentional switching between preserved non-visual modalities of the sensorium. Therefore, understanding possible changes in synaptic efficacies that might underlie functional connectivity differences in EB requires awareness of these adaptive behaviors. Persistent behavioral adaptations can strengthen synaptic efficacy in functionally connected networks (Lewis et al., 2009). Furthermore, greater correlations occur within networks serving identifiable functions. In EB, resting state fMRI differences included increased functional connectivity between visual cortex and cognitive control networks concerned with memory, attention, or task switching in frontal and parietal cortex. In contrast, there was decreased functional connectivity between visual and non-visual sensory networks and within extra-striate visual cortex. Most probably, extra-striate areas with resident multisensory inputs conveyed cross-modal inputs through existing connections with non-visual cortex. The enhancement of cross-modal activity might then result from adapting to visual deprivation.

Crucial to functional connectivity analyses is isolating brain-derived signals from artifact (Cordes et al., 2001). The current methodology relies on regression of nuisance signals, including the global signal (GSR) (Fox et al., 2009). It has been pointed out that GSR can artificially induce group differences if the global signal is unequally distributed in the two groups (Saad et al., 2012), which was not the case between the EB and NS groups.

A principal limitation of this study is that the functionality represented in the visual cortex of EB depended entirely on the extant literature reporting task-based fMRI responses to memory and attention paradigms. However, we submit that this literature is sufficiently mature to support such use. The relatively small participant sample size was limited by requiring that all EB were blind from peripheral pathologies and be Braille literate from childhood. Echo planar imaging data also had to meet standards of minimal movement (Hacker et al., 2012). A larger sample of younger participants with a range of Braille literacy may permit identification of age and literacy effects on resting-state activity.

### Conflict of interest statement

The authors declare that the research was conducted in the absence of any commercial or financial relationships that could be construed as a potential conflict of interest.

## Footnotes

^1^We use “visual cortex” as an anatomic descriptor to indicate cortex that normally is associated with visual function in sighted persons.

^2^The average signal values for the global regressor were not significantly different between the groups.

^3^We used three of four runs obtained from EB that showed the least head movement.
